# Characterisation of a hyperthermophilic transketolase from *Thermotoga maritima* DSM3109 as a biocatalyst for 7-keto-octuronic acid synthesis†

**DOI:** 10.1039/d1ob01237a

**Published:** 2021-07-07

**Authors:** Max Cárdenas-Fernández, Fabiana Subrizi, Dragana Dobrijevic, Helen C. Hailes, John M. Ward

**Affiliations:** Department of Biochemical Engineering, University College London Gower Street London WC1E 6BT UK m.cardenas-fernandez@kent.ac.uk j.ward@ucl.ac.uk; School of Biosciences, University of Kent Canterbury Kent CT2 7NJ UK; Department of Chemistry, University College London 20 Gordon Street London WC1H 0AJ UK

## Abstract

Transketolase (TK) is a fundamentally important enzyme in industrial biocatalysis which carries out a stereospecific carbon–carbon bond formation, and is widely used in the synthesis of prochiral ketones. This study describes the biochemical and molecular characterisation of a novel and unusual hyperthermophilic TK from *Thermotoga maritima* DSM3109 (TK_tmar_). TK_tmar_ has a low protein sequence homology compared to the already described TKs, with key amino acid residues in the active site highly conserved. TK_tmar_ has a very high optimum temperature (>90 °C) and shows pronounced stability at high temperature (*e.g. t*_1/2_ 99 and 9.3 h at 50 and 80 °C, respectively) and in presence of organic solvents commonly used in industry (DMSO, acetonitrile and methanol). Substrate screening showed activity towards several monosaccharides and aliphatic aldehydes. In addition, for the first time, TK specificity towards uronic acids was achieved with TK_tmar_ catalysing the efficient conversion of d-galacturonic acid and lithium hydroxypyruvate into 7-keto-octuronic acid, a very rare C_8_ uronic acid, in high yields (98%, 49 mM).

## Abstract 2

Hyperthermophilic and thermostable transketolase from *Thermotoga maritima* (TK_tmar_) catalysed the efficient conversion of d-galacturonic acid into 7-keto-octuronic acid.

## Graphical abstract

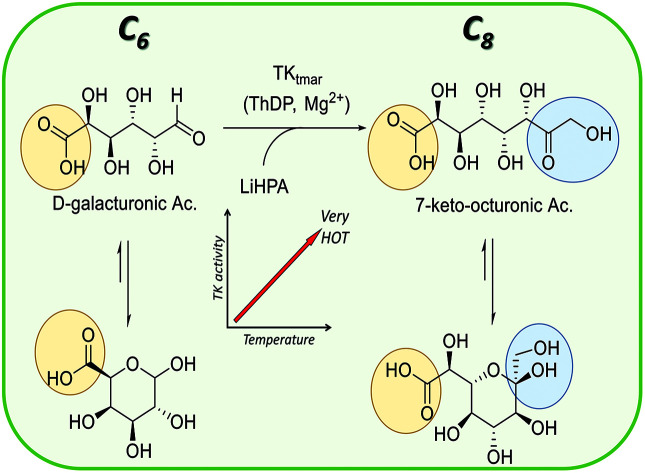

## Introduction

Biocatalysis is considered as a green and sustainable technology based on the principles and metrics of green chemistry and sustainable development.^1^ Application of enzymatic reactions in industrial chemical processes is very advantageous due to their high stereo- and regioselectivity, low energy demand and because enzymatic catalysts are renewable.^2^ In order to enhance the contribution of biocatalysis to a more sustainable society, increasing the availability of different of enzyme classes through enzyme discovery and engineering is paramount.

Transketolase (TK, EC 2.2.1.1) is a thiamine diphosphate (ThDP) dication (usually Mg^2+^) dependent enzyme, that catalyses the *in vivo* reversible carbon–carbon bond formation between d-xylulose-5-phosphate and d-ribose-5-phosphate yielding d-sedoheptulose-7-phosphate and glyceraldehyde-3-phosphate. TK follows a Ping-Pong Bi–Bi reaction kinetics where the donor and acceptor substrates are not able to bind to the protein simultaneously.^3^ TK is a key enzyme linking the pentose phosphate and the glycolysis pathways.^4,5^ From the synthetic point of view, wild-type (WT) TK adds a 2-carbon unit to an aldehyde creating a C–C bond between the aldehyde acceptor and a carbonyl or a keto acid donor substrate, and generating a new stereogenic centre. This forms a new asymmetric α-hydroxyketone with an (*S*)-configuration, which is otherwise difficult to prepare chemically.^6,7^ WT and engineered TKs from different microbial sources have been used in the synthesis of phosphorylated sugars,^8^ non-phosphorylated aliphatic and aromatic acyloins,^9–13^ and rare sugars such as l-glucoheptulose,^14,15^ as well as in coupled cascade reactions.^15–18^

**Scheme 1:**
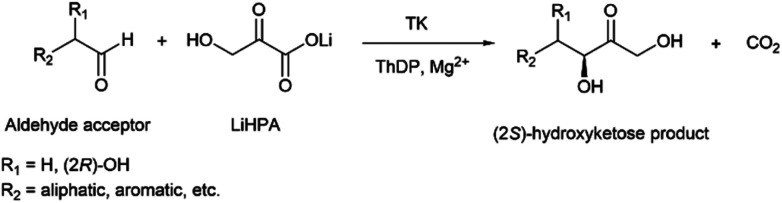
Transketolase reaction with aldehyde acceptors and lithium hydroxypyruvate (LiHPA) as carbon donor.

For synthetic applications, biocatalysis using enzymes such as TK allows a sustainable, one-step stereoselective way for preparing chiral building blocks and fine chemicals. In synthetic uses, the utilisation of lithium hydroxypyruvate (LiHPA) in TK-catalysed reactions is well established as a common ketol donor (Scheme 1). TKs can be deactivated during biocatalytic syntheses by different processes such as oxidation, substrate or product inhibition, or dissociation of the ThDP cofactor; all of this can be avoided by applying reaction engineering strategies including the use of reducing agents, substrate feeding, or *in situ* product removal.^19^ However, the use of thermostable or thermophilic enzymes also avoids some of these limitations.

Thermophilic enzymes are sourced mainly from microorganisms able to live in high temperature environments. These enzymes possess an optimum temperature above 50 °C and extended activity half-life (*t*_1/2_) at high temperatures, being more robust under demanding industrial applications such as high temperatures and the presence of organic solvents compared to their mesophilic counterparts.^20,21^*Thermotoga maritima* (DSM3109 or MSB8) is an anaerobic hyperthermophilic bacterium, which was first isolated from geothermally heated marine sediments near Vulcano Island in Italy; it has an optimum growth temperature of 80 °C and a G + C content of 46.2%.^22^*T. maritima* is able to grow on several monosaccharides and disaccharides as the sole carbon and energy source.^23^ The genome sequence of *T. maritima* revealed that a substantial portion of its genes were inherited *via* horizontal or lateral gene transfer from Archaea, and from anaerobic members of the Firmicutes, the low GC Gram-positive bacteria.^24^

To date, despite their high demand in industrial biotechnology, only a few thermostable TKs have been described. Recently a “split-gene” TK from *Carboxydothermus hydrogenoformans* (TK_chy_) has been reported showing an optimum temperature of 70 °C and good stability in the presence of different organic solvents.^25^ Also, Bawn *et al.*^15^ isolated and characterised TKs from *Deinococcus geothermalis* (TK_dge_) and *Deinococcus radiodurans* (TK_dra_) which were used to produce l-glucoheptulose from l-arabinose; and both TKs have optimum temperatures of 50 °C. Notably, TK from *Geobacillus stearothermophilus* (TK_gst_) was the first thermostable TK reported also with an optimum temperature of 50 °C.^26^ TK_gst_ has been subjected to multiple protein engineering studies in order to expand its substrate scope.^9,27–29^

In this work, we report the biochemical characterisation of a hyperthermophilic TK from *T. maritima* DSM3109, with extreme thermostability, as well as investigations into substrates accepted including C_5_ and C_6_ monosaccharides, sugar derivatives (*e.g.* glucosamine and furfural) and aliphatic aldehydes (C_3_–C_9_) using LiHPA as the ketol donor. In addition, we describe for the first time, TK-catalysed conversions of uronic acids. In particular, the efficient bioconversion of d-galacturonic acid into a rare C_8_ sugar 7-keto-octuronic acid is presented.

## Results and discussion

### TK_tmar_ protein identification and sequence analysis

TK_tmar_ protein and gene sequences were retrieved from UniProtKB (R4NSD7). The gene was 1908 bp long with a 49.6% G + C content. Although a high G + C content (>60%) is associated with thermal adaptation, this is not necessary the case for all thermophilic prokaryotes.^30,31^ Several factors have been associated with protein thermostability such as certain amino acid substitutions, hydrophobic cores, buried polar contacts and ion pairs and interactions amongst subunits.^32^ TK_tmar_ is homodimeric, composed of 635 amino acid residues and has a molecular mass of 69.9 kDa per monomer (as shown in SDS-PAGE after Ni-NTA chromatography purification, ESI Fig. 1†) with a theoretical pI of 5.6 (http://web.expasy.org/compute_pi/). The amino acid composition is considered as the primary factor for protein thermostability prediction. Comparing the protein sequence of TK_tmar_ to other well-characterised TKs from *G. stearothermophilus*, *E. coli*, *B. anthracis*, *S. cerevisiae*, *H. sapiens*, *C. hydrogenoformans* and *S. oleracea*; TK_tmar_ has a higher content in charged (Asp and Lys) and hydrophobic side chain (Val and Tyr) amino acid residues. TK_tmar_ also has a lower frequency of polar uncharged amino acids residues (Gln, Ser and Thr). All of these properties are associated with an improved protein thermostability.^32,33^ TK_tmar_ has a very low Ala content (7% compared to the 11% average) and 5 Cys residues (compared to only 1 for *G. stearothermophilus* and *C. hydrogenoformans*) that could potentially be forming disulfide bonds as a mechanism for protein stabilisation (ESI Table 1†).^34^ It has been suggested that Pro residues in the loop regions play an important role in enzyme thermostability;^25,35^ however, TK_tmar_ contains only 26 Pro residues (4.1%), which together with *B. anthracis* has the lowest number of Pro residues, amongst the analysed TKs.

**Fig. 1:**
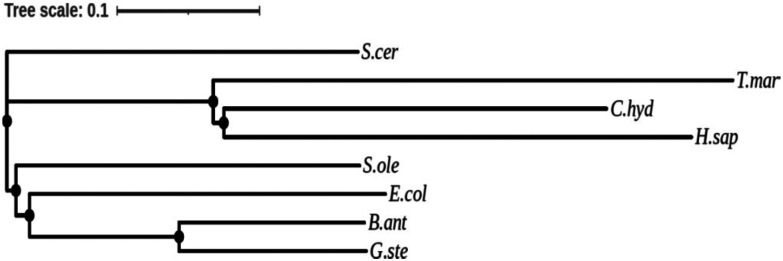
Phylogenetic analysis of TKs were generated with Clustal Omega and formated using the iTOL server (http://itol.embl.de/index.shtml). *T. maritima* (T.mar), G*. stearothermophilus* (G.ste), *E. coli* (E.col), *B. anthracis* (B.ant), *S. cerevisiae* (S.cer), *H. sapiens* (H.sap), *C. hydrogenoformans* (C.hyd) and *S. oleracea* (S.ole).

Multiple sequence alignment (ESI Fig. 2†) and phylogenetic analysis (Fig. 1), showed a low homology between TK_tmar_ and other TKs, with a higher similarity to TK_chy_ (35.7%) and TK from *H. sapiens* (30.2%) (ESI Table 2†). In spite of their low sequence identity, 66 amino acid residues are highly conserved amongst all TKs, including the key ones in the active site,^36^ except for (based on *E. coli* numbering) Gly156 (ThDP binding), Ile/Leu187 (metal ion binding) and His473 (substrate binding) which are replaced by Ala, Ala and Gln in TK_tmar_, respectively.

### Biochemical characterisation

**Fig. 2:**
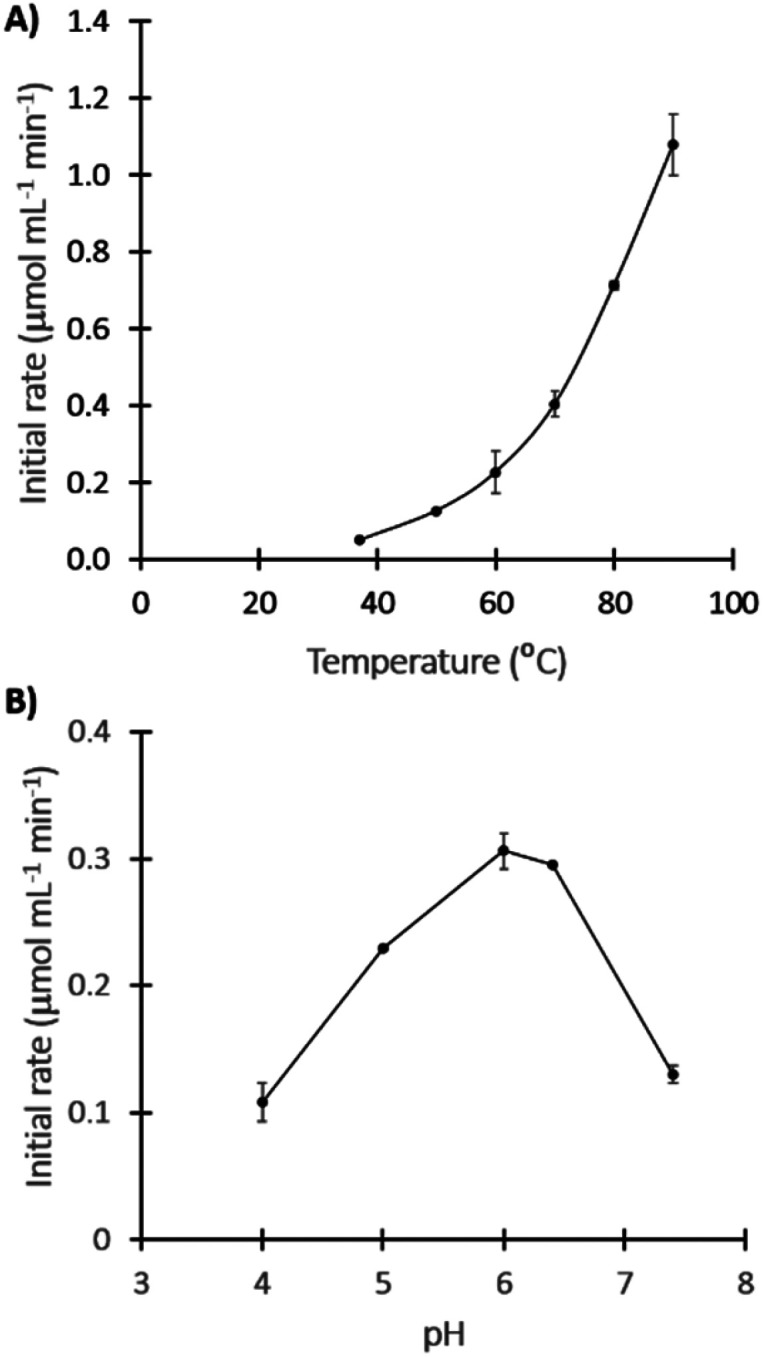
Determination of TK_tmar_ optima activity conditions. (A) Temperature, in TRIS-HCl 0.1 M buffer pH 7. (B) pH, performed at 60 °C in acetate 0.1 M buffer (pH 4, 5 and 6) or TRIS-HCl 0.1 M buffer (pH 6.4 and 7.4). All experiments were carried out in duplicate.

TK_tmar_ optima temperature and pH were determined by measuring the initial rates (for up to 20 min) of the reaction between glycolaldehyden (GA) and LiHPA to yield l-erythrulose (Ery). The optimum temperature was not possible to estimate based on our set of experiments (max 90 °C), inferring that the TK_tmar_ optimal temperature might be around or above the boiling point of water. Notably, the initial rate at 90 °C was 21.7-fold higher compared to the one at 37 °C (Fig. 2A). Most hyperthermophilic enzymes are optimally active at temperatures close to the host organism's optimum growth temperature; however, it has also been reported that some hyperthermophilic enzyme have an optimum temperature above this value, or even above the boiling point of water.^33,37^ In addition, the Ery yield after 20 min of reaction also increased with temperature, achieving around 76% at 80 and 90 °C, 8-fold higher compared to at 37 °C (ESI Fig. 3†).

The pH optimum was found between 6 and 6.4 which is below the optima pH reported for other thermophilic TKs (pH 7 and 8);^15,25,26^ in fact, TK_tmar_ lost 57% of its activity at pH 7.4 compared to pH 6.4 (Fig. 2B).

Apparent kinetic parameters were calculated following the pH-based high-throughput assay with GA and LiHPA as substrates at pH 7 and 65 °C (maximum equipment temperature) (Table 1, ESI Fig. 4†). *K*_M_ calculations indicated that TK_tmar_ has a higher affinity towards GA (the aldehyde acceptor) than towards LiHPA, in contrast to other TKs that show more affinity towards the ketol donor (LiHPA) as reported using the same activity assay.^38^ Likewise, *k*_cat_ and *k*_cat_/*K*_M_ values showed a similar trend and they were significantly lower compared to that reported for other TKs.^38^

**Table 1:** TK_tmar_ apparent kinetic parameters towards LiHPA and glycoaldehyde (GA), were calculated following the pH-based high-throughput assay with pure TK_tmar_ (0.25 mg mL^−1^), GA (final concentration 0.5 to 50 mM – LiHPA constant at 50 mM) or LiHPA (final concentration 5 to 100 mM – GA constant at 50 mM), phenol red (28 mM), ThDP (2.4 mM), MgCl_2_ (9 mM). All reaction components were prepared in TEA 2 mM buffer pH 7 and reactions carried out 65 °C. The reactions were monitored at 560 nm for up to 30 min in a plate reader. *K*_M_ and *V*_max_ were determined with OriginPro 2018 software. All experiments were carried out in duplicate

	*V*_max_	K_M_	*k*_cat_	*k*_cat_/*K*_M_
	(mM min^−1^)	(mM)	(s^−1^)	(mM^−1^ s^−1^)
LiHPA	0.33 ± 0.01	53.8 ± 4.1	0.77 ± 0.03	0.0144 ± 0.001
GA	0.19 ± 0.01	3.6 ± 0.4	0.45 ± 0.03	0.1257 ± 0.006

### Stability of TK_tmar_

**Fig. 3:**
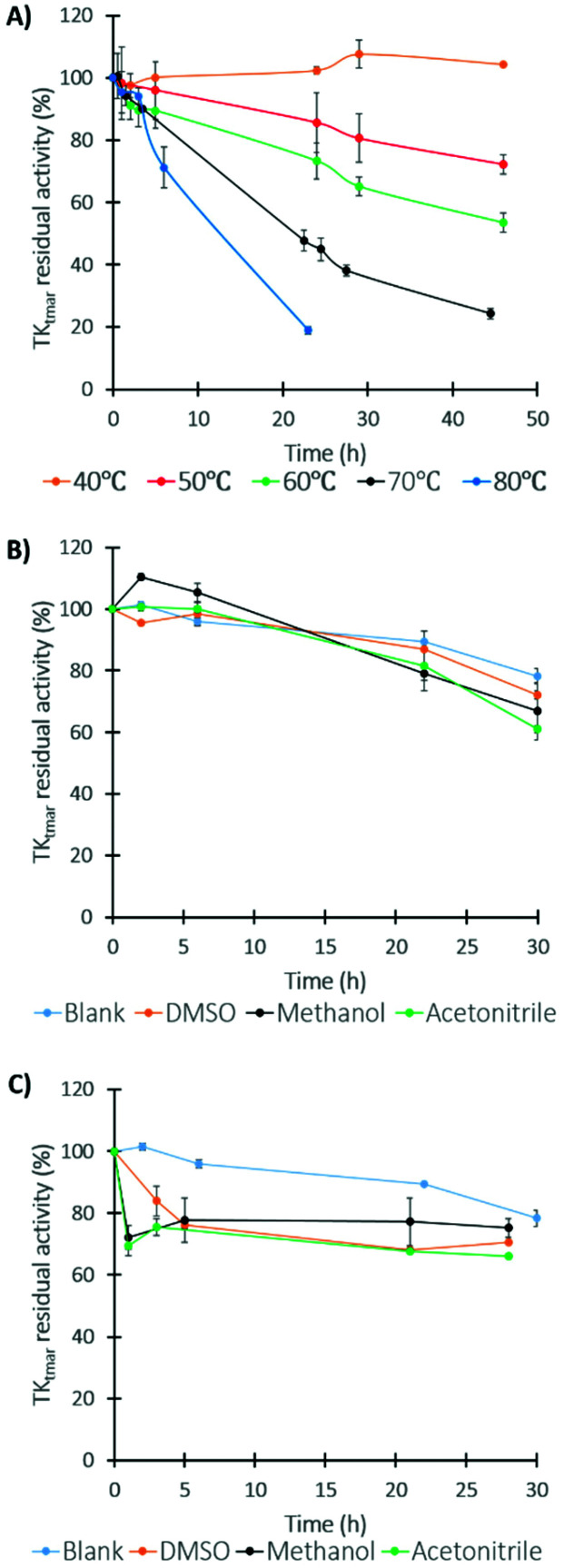
TK_tmar_ stability characterisation. (A) Thermal stability, (B) organic solvent stability 10% (v/v) and (C) organic solvent stability 50% (v/v). All experiments were performed with TK_tmar_ (0.7 U mL^−1^), ThDP (2.4 mM) and MgCl_2_ (9 mM) in TRIS-HCl 0.1 M buffer pH 7. Organic solvent stability experiments were performed at 50 °C. Enzymatic activity was measured at pH 7.4 and 40 °C following TK_tmar_ enzymatic assay. All experiments were carried out in duplicate.

Enzyme stability was determined by incubating purified TK_tmar_ under different conditions. Samples were taken periodically, cooled down and then initial rates were calculated following the TK activity assay with Ery and d-ribose-5-phosphate as substrates. TK_tmar_ did not lose activity at 40 °C after 45 h. Despite the loss of activity, which was observed with temperature increase, TK_tmar_ was shown to be highly thermostable with *t*_1/2_ of 99, 54.1, 21.2 and 9.3 h at 50, 60, 70 and 80 °C, respectively (Fig. 3A). This is a remarkable feature for TK_tmar_ compared to TK_gst_ that was fully deactivated at 70 °C after 1 h.

Thermophilic enzymes have been shown to be more robust and suited to demanding chemical processes such as those utilising organic solvents.^20,21^ TK_tmar_ stability in three organic solvents commonly used in industry (DMSO, methanol and acetonitrile) was tested. The data in Fig. 3B and C shows that TK_tmar_ is highly stable at low (10% v/v) and high (50% v/v) organic solvent concentrations for up to 30 h, retaining on average 67% and 70% of the initial activity respectively. When the enzyme was incubated in presence of 10% (v/v) organic solvent, the loss of activity profile was similar to the control reaction; interestingly, a more rapid loss of activity during the first hours of incubation was observed at 50% (v/v) of organic solvent but followed by activity stabilisation. TK_tmar_ showed better organic solvent stability properties compared to TK_chy_ whose loss of activity was around 50% after 1 h incubation in the same organic solvents.^25^

### Substrate scope screening

The TK pH-based high-throughput assay proved to be effective for initial substrate scope screening for a series of aldehydes substrates using LiHPA as ketol donor.^9,38^ In this work several aldehyde donors were tested including monosaccharides: C_5_ (l-rhamnose, l-arabinose, d-ribose, 2-deoxy-d-ribose and d-xylose); C_6_ (d-glucose, d-galactose and d-mannose); uronic acids (d-galacturonic acid and d-glucoronic acid); and monosaccharide derivatives (d-glucosamine and 5-hydroxyfurfural); as well as aliphatic aldehydes (C_3_ to C_9_). The initial reaction rates were compared to that for the GA reaction.

**Fig. 4:**
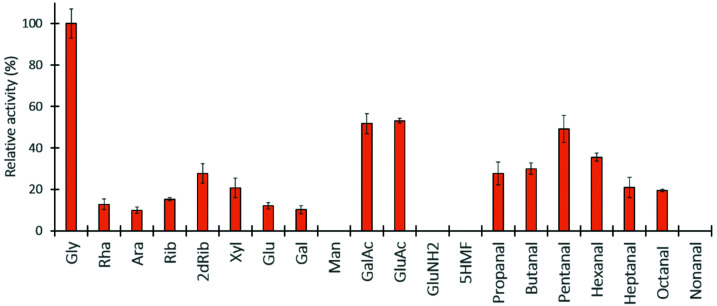
TK_tmar_ substrate scope screening. Relative activity towards several aldehyde substrates (with glycoaldehyde (GA) as 100%) and LiHPA as carbon donor was calculated following the pH-based high-throughput assay with pure TK_tmar_ (0.25 mg mL^−1^) and pH 7.2 at 50 °C. Reactions were performed with 200 mM of monosaccharides: l-rhamnose (Rha), l-arabinose (Ara), d-ribose (Rib), 2-deoxy-d-ribose (2dRib), d-xylose (Xyl), d-glucose (Glu), d-galactose (Gal), d-mannose (Man), d-galacturonic acid (GalAc), d-glucoronic acid (GluAc), d-glucosamine (GluNH_2_), 5-hydroxyfurfural (5HMF). Aliphatic aldehydes (propanal, butanal, pentanal, hexanal, heptanal, octanal and nonanal) were tested at 100 mM with addition of DMSO (20%). All experiments were carried out in duplicate.

It has been described that TKs can accept both non-phosphorylated and phosphorylated substrates, showing higher affinity for the latter (natural substrates).^18^ Overall, TK_tmar_ exhibited low activity towards both C_5_ and C_6_ monosaccharides with a relative activity below 30% (Fig. 4). No activity was observed towards d-mannose and the C_6_ derivatives. Our results are in agreement with those ones published by Yi *et al.* where TK_gst_ was tested with similar non-phosphorylated monosaccharides.^38^ However, Bawn *et al.* showed that TK_dge_ and TK_dra_ accepted l-arabinose which was upgraded to l-glucoheptulose in high yields.^15^ TK_tmar_ also showed activity towards C_3_ to C_8_ aliphatic aldehydes, with the highest relative activity for pentanal (49%). No activity was detected towards nonanal. Likewise, TK_gst_ had a very similar profile activity towards these substrates and its activity towards heptanal and octanal was very low.^9^

More interestingly, our results showed that TK_tmar_ is active towards uronic acids with relative activities towards d-galacturonic acid (GalAc) and d-glucoronic acid of 52% and 53% respectively. This TK-catalysed reaction would lead to the formation of the respective 7-keto-octuronic acids.

Eight-carbon monosaccharides or octuloses are rare in nature being naturally produced by some plant species and by a few microorganisms but in low concentrations.^39^ However, TK catalyses the C–C bond formation between d-xylulose-5-phosphate and d-ribose-5-phosphate yielding a C_7_ monosaccharide d-sedoheptulose-7-phosphate.^40,41^ In addition, it has been suggested that octulose-phosphates are synthesised in the non-oxidative pentose phosphate pathway catalysed by a TK; for example, TK from spinach catalysed d-*glycero*-d-ido-octulose-8-phosphate synthesis from fructose-6–phosphate and glucose-6-phosphate *in vivo*.^42^ To the best of our knowledge, this is the first study where the synthesis of 7-keto-octuronic acids catalysed by a TK has been definitively reported.

### 7-Keto-octuronic acid preparative synthesis and identification

**Scheme 2:**
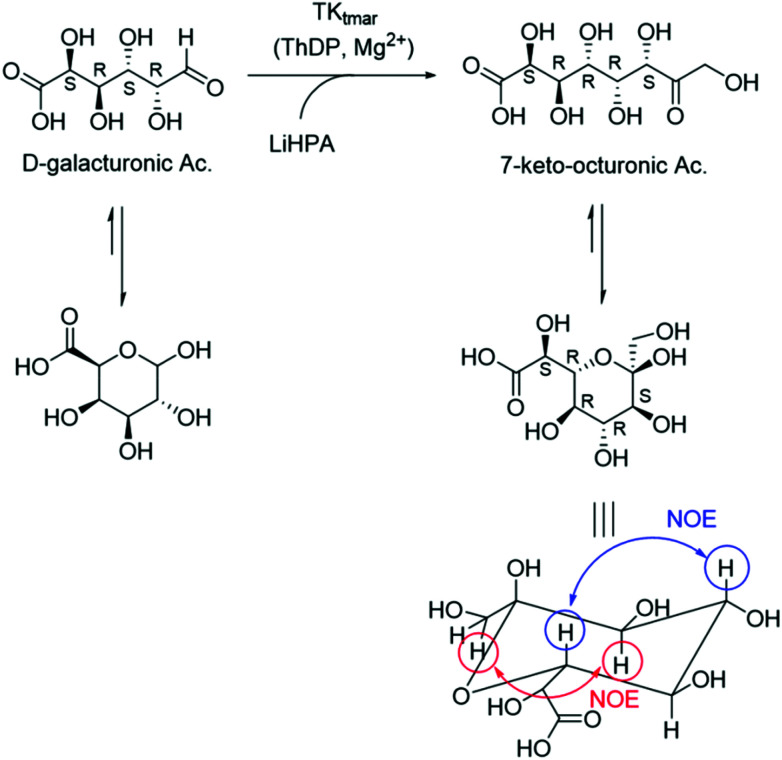
TK_tmar_ catalysed the high-yield synthesis of 7-keto-octuronic acid from d-galacturonic acid and lithium hydroxypyruvate (LiHPA) as carbon donor.

To demonstrate the utility of this new reactivity towards uronic acids, the preparative scale synthesis of (2*S*,3*R*,4*R*,5*R*)-2,3,4,5,6,8-hexahydroxy-7-oxo-octanoic acid (7-keto-octuronic acid, OctAc) was carried out on a 25 mL reaction volume using GalAc and LiHPA as starting materials (Scheme 2). GalAc is a relatively inexpensive chemical, which is mainly obtained from the hydrolysis of pectin renewable biomass, for example sugar beet pulp.^43,44^ The reaction was performed at 50 °C to avoid LiHPA degradation that occurs at higher temperatures, particularly for longer reaction times.^16^ By ICS analysis, 84% of GalAc was converted in to OctAc after a 1 h reaction. In order to increase GalAc conversions, 1 mL of LiHPA 50 mM (final concentration 20 mM) was added after 4.5 h reaction and this strategy increased the GalAc conversion to 98%, equivalent to 49 mM of OctAc being produced.

OctAc was then isolated by a Dowex 50WX8 anion exchange column first, to remove excess of TRIS buffer, to give OctAc with a final isolated yield of 90%. ^1^H NMR spectroscopy confirmed that OctAc was formed with the major product in the pyranose form (ESI Fig. 5†). In the TK reaction, a new stereogenic centre was formed and considering the 2 possible anomers of the cyclic pyranose form, a mixture of 4 different isomers can be generated.

Remarkably, NMR spectroscopy of a freshly prepared sample identified the presence of one major isomer in the form of pyranose. The large *trans* coupling constant between the axial protons at 5-H and 6-H also confirmed that the hydroxyl group at C-6 was in an equatorial orientation giving an *S*-configuration to the newly generated stereocentre at this position. The TK catalysed reaction showed exceptional diastereoselectivity leading to a ketose with the d-*threo* configuration, which is consistent with the stereoselectivity observed in TK reactions with other aldose sugars.^6^ The major anomer was identified to be the more stable α-anomer; however, OctAc exhibited mutarotation behaviour; a solution of the acid in D_2_O was analysed by NMR spectroscopy after several days showing an increasing amount of the other isomers (see ESI Fig. 6† for comparison ^1^H NMR analyses after 2 and 4 weeks).

## Experimental

### Materials

All chemicals were purchased from Merck KGaA (Sigma-Aldrich) and molecular biology reagents were from New England Biolabs, unless otherwise stated. LiHPA was prepared as previously reported.^45^

### Transketolase cloning, expression and purification

The protein sequence of the putative transketolase from *Thermotoga maritima* DSM3109 (TK_tmar_) was retrieved from UniProtKB (ID number: R4NSD7). TK_tmar_ cloning was carried out with the modified plasmid pET-29:SacB-SapI as previously described.^46^ PCR primers were designed by adding overhangs homologous to the pET-29:SacB-SapI vector; forward: 5′-AAAGCTCTTCGATGGAAAGGTTTCCTATGA-3′ (*T*_m_ = 60.3 °C) and reverse: 5′-AAAGCTCTTCGGTGGAGCATCTCTCTGAGTCTGG-3′ (*T*_m_ = 57.7 °C). TK_tmar_ gene was amplified using Phusion® High-Fidelity PCR Master Mix, 100 ng of genomic *T. maritima* DNA, 0.5 μM of each primer and DMSO 5% in 50 μL reaction. PCR was carried out as follow: initial denaturation, 98 °C for 3 min; 30 cycles of denaturation 98 °C for 10 s, annealing 58 °C for 30 s, extension 72 °C for 45 s; and final extension, 72 °C for 10 min. The amplicon was confirmed by agarose gel electrophoresis (1% in TBE buffer) and PCR products were then recovered from the gel following Monarch® DNA Gel Extraction Kit protocol. TK_tmar_ cloning was performed with a one-pot restriction-ligation reaction method;^46^ transformed into chemically competent *E. coli* NovaBlue that were plated on LB agar supplemented with sucrose 20% and kanamycin 50 μg mL^−1^ and grown at 37 °C overnight. Colonies were picked and then grown overnight in 10 mL of LB broth with kanamycin 50 μg mL^−1^ at 37 °C and 250 rpm, and plasmids were extracted using QIAprep Spin Miniprep kit (Qiagen) and sent for sequencing (Eurofins Genomics) to confirm gene sequences. Positive plasmid construct was then transformed into expression host *E. coli* BL21(DE3).

For expression, recombinant TK_tmar_ (containing a *C*-terminal His_6_Tag) was grown in a 2 L shake-flask with 200 mL of TB broth with glycerol 8 mL L^−1^, kanamycin 50 μg mL^−1^ at 37 °C and 250 rpm. IPTG was added (0.1 mM final concentration) when the OD_600 nm_ reached ∼1.5, then kept at 25 °C and 250 rpm for 15 h. Cells were recovered by centrifugation (10 000 rpm at 4 °C for 30 min) and the cell pellet resuspended in TRIS-HCl 50 mM buffer pH 7 (cell concentration 0.5 g mL^−1^) and then lysed by sonication on ice (10 s on and 15 s off for 25 cycles) followed by centrifugation (12 500 rpm at 4 °C for 30 min). The clarified lysate was kept at 4 °C (or −20 °C for up to one month) for further analysis.

TK_tmar_ was purified by IMAC methodology with a 5 mL Ni-NTA agarose column (Qiagen) following the manufacturer's instructions. Clarified lysate (prepared in 10 mM imidazole buffer) was loaded into pre-equilibrated column (10 mM imidazole buffer), then the column washed (50 mM imidazole buffer) and finally the enzyme was eluted with 0.5 M imidazole buffer. TK_tmar_ was finally precipitated with ammonium sulfate 70% saturation and kept at 4 °C. All imidazole solution buffers were prepared in TRIS-HCl 50 mM with NaCl 0.3 M and pH adjusted to 7.

Enzyme expression and purification was confirmed with SDS-PAGE analysis with Novex® TBE 10% gels (Invitrogen) and NuPAGE™ MOPS-SDS running buffer (Thermofisher Scientifc). Protein quantification was carried with Quick Start Bradford Protein Assay (Bio-Rad) using bovine serum albumin as protein standard.

### Transketolases phylogenetic analysis

TKs sequences from different sources were retrieved from UniProtK: *Geobacillus stearothermophilus* (A0A0I9QGZ2), *Escherichia coli* (P27302.5), *Bacillus anthracis* (Q81Y15), *Saccharomyces cerevisiae* (P23254), *Homo sapiens* (P29401), *Carboxydothermus hydrogeoformans* (Q3AFP7 and Q3AFP6) and *Spinacia oleracea* (O20250). Multiple sequence alignment and phylogenetic analysis were carried out using Clustal Omega, Jalview and MEGA 10. iTOL server was used for phylogenetic tree visualisation and formatting.

### Transketolase characterisation

All pHs values for TRIS-HCl and triethanolamine (TEA) buffers were adjusted for 37 °C. Final experimental pH value at higher temperatures were estimated by considering a temperature coefficient of −0.028 and −0.02 per °C, respectively.

#### Biochemical characterisation

TK_tmar_ optima temperature and pH were determined by measuring the initial rates (for up to 20 min) of the reaction with GA and LiHPA to yield Ery. The reaction contained GA (10 mM), LiHPA (10 mM), ThDP (2.4 mM), MgCl_2_ (9 mM) and TK_tmar_ (0.05 mg mL^−1^). For optimum temperature experiments, the reaction was carried out in TRIS-HCl 50 mM buffer pH 7 and incubated at 37, 50, 60, 70, 80 or 90 °C. Optimum pH experiments were performed at 60 °C in 0.1 M buffer acetate (pH 4, 5 and 6) or TRIS-HCl (pH 6.4 and 7.4). All experiments were carried out in duplicate, 1 mL reaction volume and in a Eppendorf Thermomixer™ C fitted with a Thermotop (to avoid condensation on tube lid) with agitation at 750 rpm. Samples were taken periodically, mixed with one volume of TFA 0.5% (v/v) and then analysed by HPLC.

#### Determination of apparent kinetics parameters

Kinetic parameters were calculated following the pH-based high-throughput assay described by Yi *et al.*^38^ 10 μL of pure TK_tmar_ (stock 5 mg mL^−1^) was added to 150 μL of reaction mixture containing phenol red (28 μM), ThDP (2.4 mM), MgCl_2_ (9 mM), and GA (final concentration 50, 25, 10, 5, 2.5, 1 or 0.5 mM), this mixture was preincubated at 65 °C for 10 min followed by the addition of 40 μL of preincubated LiHPA (final concentration 50 mM). A similar procedure was performed keeping GA (50 mM) concentration constant and varying LiHPA concentration (final concentration 100, 75, 50, 25, 12.5 or 5 mM). All reaction components were prepared in TEA 2 mM buffer pH 7. The reactions were carried out in duplicates and monitored at 560 nm for up to 30 min in a plate reader. Linear regression (*R*^2^ > 0.95) was used to measure the slope of the initial rates. NaHCO_3_ (1, 0.75, 0.5, 0.25, 0.1 and 0.05 mM) standard curve was constructed and used for enzyme activity calculation expressed as μmol mL^−1^ min^−1^. *K*_M_ and *V*_max_ were determined with OriginPro 2018 software.

#### Stability studies

TK_tmar_ thermostability was evaluated by incubating pure TK_tmar_ (0.7 U mL^−1^), ThDP (2.4 mM) and MgCl_2_ (9 mM) in TRIS-HCl 0.1 M buffer pH 7 at 40, 50, 60, 70 and 80 °C for up to 45 h. Likewise, co-solvent stability was performed at similar conditions and in presence of 10 or 50% (v/v) of either DMSO, acetonitrile or methanol and incubated at 50 °C for up to 30 h. Sample were taken periodically, cooled down in an ice bath for 5 min, and the enzymatic activity was measured following the TK_tmar_ enzymatic assay.

#### Substrate scope screening

Activity towards several monosaccharides and aliphatic aldehydes as carbon acceptors was screened using the pH-based assay as described above and in buffer TEA 2 mM pH 7.2 at 50 °C. Reactions were performed with 200 mM of monosaccharides: l-rhamnose, l-arabinose, d-ribose, 2-deoxy-d-ribose, d-xylose, d-glucose, d-galactose, d-mannose, d-galacturonic acid (GalAc), d-glucoronic acid; sugar derivatives: d-glucosamine, 5-hydroxyfurfural. Aliphatic aldehydes (propanal, butanal, pentanal, hexanal, heptanal, octanal and nonanal) were also tested with this method at 100 mM final concentration with addition of DMSO 20% (v/v).

### Preparative synthesis of 7-keto-octuronic acid and characterisation

7-Keto-octuronic acid (OctAc) synthesis was carried out with GalAc (50 mM), LiHPA (10 mM), ThDP (2.4 mM), MgCl_2_ (9 mM) and TK_tmar_ clarified lysate 20% (v/v, 11.5 mg total protein per mL, TK_tmar_ represents approximately the 25% of total protein based in densitometry analysis) in TRIS-HCl 50 mM buffer pH 6.6 in a 25 mL total reaction volume at 50 °C and with moderate agitation with a magnetic stirrer. 1 mL of LiHPA 0.5 M was added after 4.5 h and the reaction was left at same conditions for up to 20 h. The reaction mixture was centrifuged and the supernatant kept at −20 °C until analysed. For product purification, an aliquot was taken (7 mL) and the pH was adjusted to 4 with HCl 1 M, then the solution was loaded on to a Dowex 50WX8 ion exchange resin (8 mL) and gently mixed for 20 min in order to remove the TRIS buffer. After this time, the eluent was collected, and the resin washed with further 3 mL of MilliQ water. All the fractions were collected and loaded onto an Ambersep 900 (OH) ion exchange resin (6 mL) and gently mixed for 30 min. The resin was washed several times with MilliQ water (2 × 7 mL) to remove salt excess. The OctAc product was then eluted with HCl 2 M (2 × 5 mL) and the resin washed with MilliQ water (5 mL). For each treatment, the mixture was agitated for 5 min prior to elution. The eluent was concentrated under reduced pressure and freeze-dried to give the OctAc as a pale brown residue (77.1 mg, 90%). Mp 73 ± 2 °C; ^1^H NMR (CD_3_OD; 600 MHz) 4.48 (1H, d, *J* = 1.5 Hz, 2-H), 4.11 (1H, dd, *J* = 10.0, 1.5 Hz, 3-H), 3.71 (1H, t, *J* = 9.4 Hz, 5-H), 3.66 (1H, d, *J* = 11.2 Hz, 8-*H*H), 3.53 (1H, dd, *J* = 10.0, 9.1 Hz, 4-H), 3.50 (1H, d, *J* = 9.6 Hz, 6-H), 3.35 (1H, d, *J* = 11.2 Hz, 8-H*H*); ^13^C NMR (CD_3_OD; 126 MHz) 175.1 (C-1), 99.2 (C-7), 75.6 (C-5), 74.6 (C-3), 71.3 (C-6), 70.8 (C-2), 70.4 (C-4), 65.0 (C-8); *m*/*z* HRMS (ES+) [MNa]^+^ 277.0528, C_8_H_14_O_9_Na requires 277.0536.

### Enzyme activity assay

TK_tmar_ activity assay was adapted to a microplate format (200 μL reaction volume per well) following a procedure previously reported by Hecquet *et al.*^47^ The reaction mixture contained Ery 100 mM, d-ribose-5-phosphate (9.1 mM), ThDP (0.1 mM), MgCl_2_ (0.5 mM), NADH (0.2 mM), alcohol dehydrogenase from *Saccharomyces cerevisiae* (25 U mL^−1^) and the TK_tmar_ suspension (10 μL, diluted when necessary). The reaction was carried out in TRIS-HCl 0.1 M buffer pH 7.4 at 40 °C, and monitored at 340 nm for up to 10 min in a plate reader (CLARIOstar Plus, BMG Labtech) and the initial rate was then calculated. One unit (U) is defined as the amount of TK_tmar_ that catalyses the formation of 1 μmol of glyceraldehyde per minute at 40 °C at pH 7.4 (value εNADH 340 nm = 6220 M^−1^ cm^−1^).

### Analytical HPLC

LiHPA and Ery were analysed by HPLC with a Ultimate 3000 + HPLC (Thermofisher Scientific) fitted with a Aminex HPX-87H column (BioRad), TFA 0.1% as mobile phase at 0.6 mL min^−1^ for 20 min and compounds detected by RI (RefractoMax 520); retention times were 8.3 and 11.5 min respectively. GalAc was analysed using an Ion Chromatography System (ICS 5000+, Thermo Scientific) using 5% (v/v) of 1 M sodium acetate (electrochemical detection grade, Fisher Scientific) with 3.8 min retention time as described by Ward *et al.*^48^ All quantitative analyses were performed measuring peak area using the external standard method.

## Conclusions

TK_tmar_ is a hyperthermophilic enzyme with unique features compared to previously reported TKs. It has a low protein homology related to well-known TKs, with 10% of amino acid residues highly conserved especially the ones in the active site. It has a high content of charged and hydrophobic side chain amino acid residues both associated with improved protein thermostability. TK_tmar_ characterisation confirmed its remarkable stability at very high temperature (*e.g. t*_1/2_ of 9.3 h at 80 °C) as well as in presence of high concentrations of organic solvents (up to 50% v/v) commonly used in the chemical industry. TK_tmar_ is able to accept a broad range of aldehyde substrates including C_5_ and C_6_ non-phosphorylated monosaccharides and aliphatic aldehydes (C_3_ to C_8_). For the first time, we have also reported that a TK can readily accept uronic acids. Preparative scale reaction from GalAc and LiHPA enabled the synthesis of 7-keto-octuronic acid with a high reaction yield (98%) and concentration of 49 mM. This novel TK_tmar_ is an excellent alternative to the current mesophilic and thermostable TKs reported. However, more studies regarding the molecular structure are needed to better understand the TK_tmar_ mechanism and how it withstands very high temperatures, as well as to expand its substrate scope further.

## Conflicts of interest

The authors declare that they have no conflict of interests.

## Supplementary Material

OB-019-D1OB01237A-s001
