# Supplementary material for: Characterisation of a hyperthermophilic transketolase from Thermotoga maritima DSM3109 as a biocatalyst for 7-keto-octuronic acid synthesis
Source: Org Biomol Chem. 2021 Jul 7;19(29):6493–500. doi: 10.1039/d1ob01237a (PMC8317047; doi:10.1039/d1ob01237a)
Supplement: OB-019-D1OB01237A-s001 [file OB-019-D1OB01237A-s001.pdf]

Electronic Supplementary Material (ESI) for Organic & Biomolecular Chemistry.
This journal is © The Royal Society of Chemistry 2021

Characterisation of a Hyperthermophilic Transketolase from *Thermotoga maritima* DSM3109 as a Biocatalyst for 7-keto-Octuronic Acid Synthesis

Max Cárdenas-Fernández[a,b]*, Fabiana Subrizi[c], Dragana Dobrijevic[a], Helen C. Hailes[c] and John M. Ward[a]*.

[a] Department of Biochemical Engineering, University College London, Gower Street, London WC1E 6BT, UK

[b] School of Biosciences, University of Kent, Canterbury, Kent CT2 7NJ, UK

[c] Department of Chemistry, University College London, 20 Gordon Street, London WC1H 0AJ, UK

* Corresponding authors: m.cardenas-fernandez@kent.ac.uk and j.ward@ucl.ac.uk

Supplementary Figure 1. Purification of TK_{tmar} and molecular weight (~70 kDa per monomer).

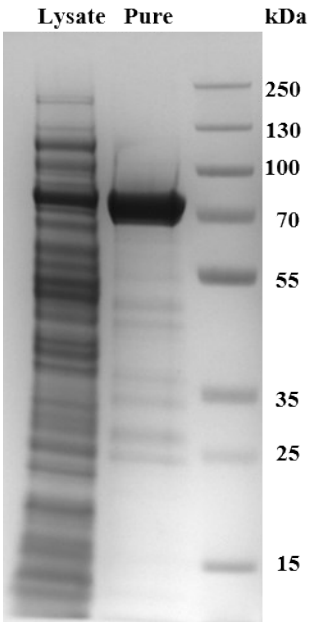

Supplementary Figure 2. Multiple sequence alignment of TKs, using Clustal Omega and Jalview bioinformatic software. Top numbers relate to TK from *E. coli*. *T. maritima* (T.mar), *G. stearothermophilus* (G.ste), *E. coli* (E.col), *B. anthracis* (B.ant), *S. cerevisiae* (S.cer), *H. sapiens* (H.sap), *C. hydrogenoformans* (C.hyd) and *S. oleracea* (S.ole).

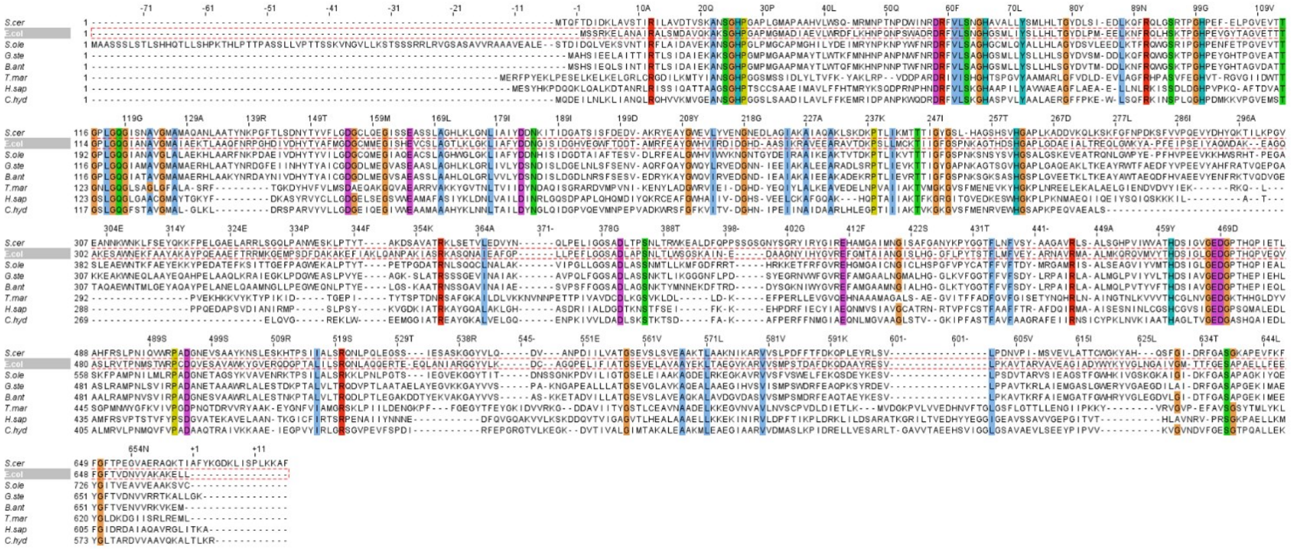

Supplementary Figure 3. L-erythrulose reaction yield (20 min) at different temperatures**,** glycoaldehyde (10 mM) and LiHPA (10 mM), ThDP (2.4 mM), $MgCl_2$ (9 mM) and TK_{tmar} (0.05 mg mL^{-1}) in TRIS-HCl 0.1 M buffer pH 7. All experiments were carried out in duplicate.

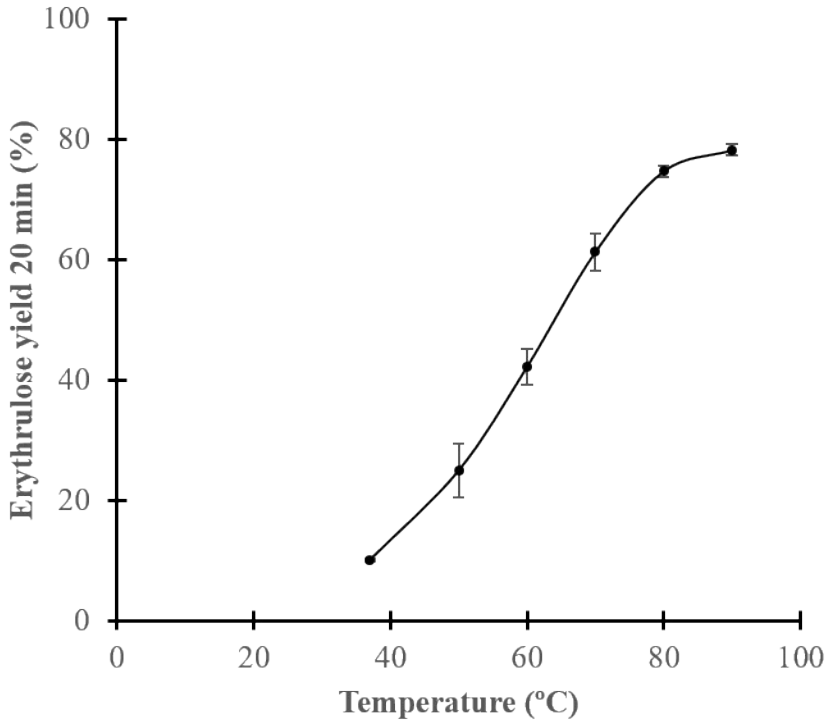

Supplementary Figure 4. TK_{tmar} kinetic parameters towards LiHPA and Glycoaldehyde, were calculated following the pH-based high-throughput assay with pure TK_{tmar} (0.25 mg mL^{-1}), glycoaldehyde (final concentration 0.5 to 50 mM - LiHPA constant at 50 mM) or LiHPA (final concentration 5 to 100 mM - glycoaldehyde constant at 50 mM), phenol red (28 μM), ThDP (2.4 mM), $MgCl_2$ (9 mM). All reaction components were prepared in 2 mM TEA buffer pH 7 and reaction carried out 65°C. The reactions were carried out in duplicates and monitored at 560 nm for up to 30 min in a plate reader. K_M and V_{max} were determined with OriginPro 2018 software.

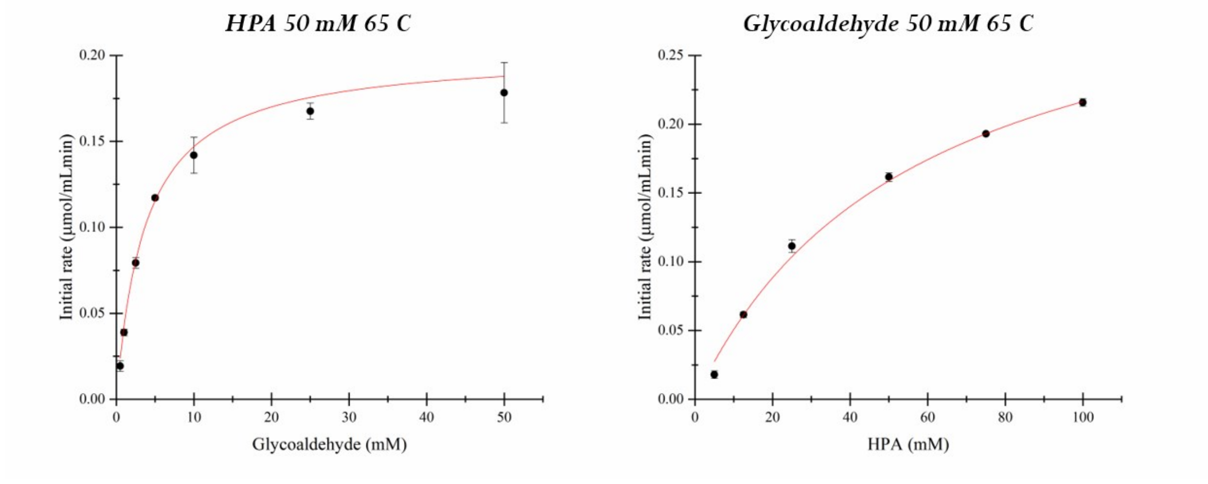

Supplementary Figure 5. ^{1}H NMR spectra (600 MHz; D_2O) for (2*S*,3*R*,4*R*,5*R*)-2,3,4,5,6,8-hexahydroxy-7-oxo-octanoic acid (7-keto-octuronic acid).

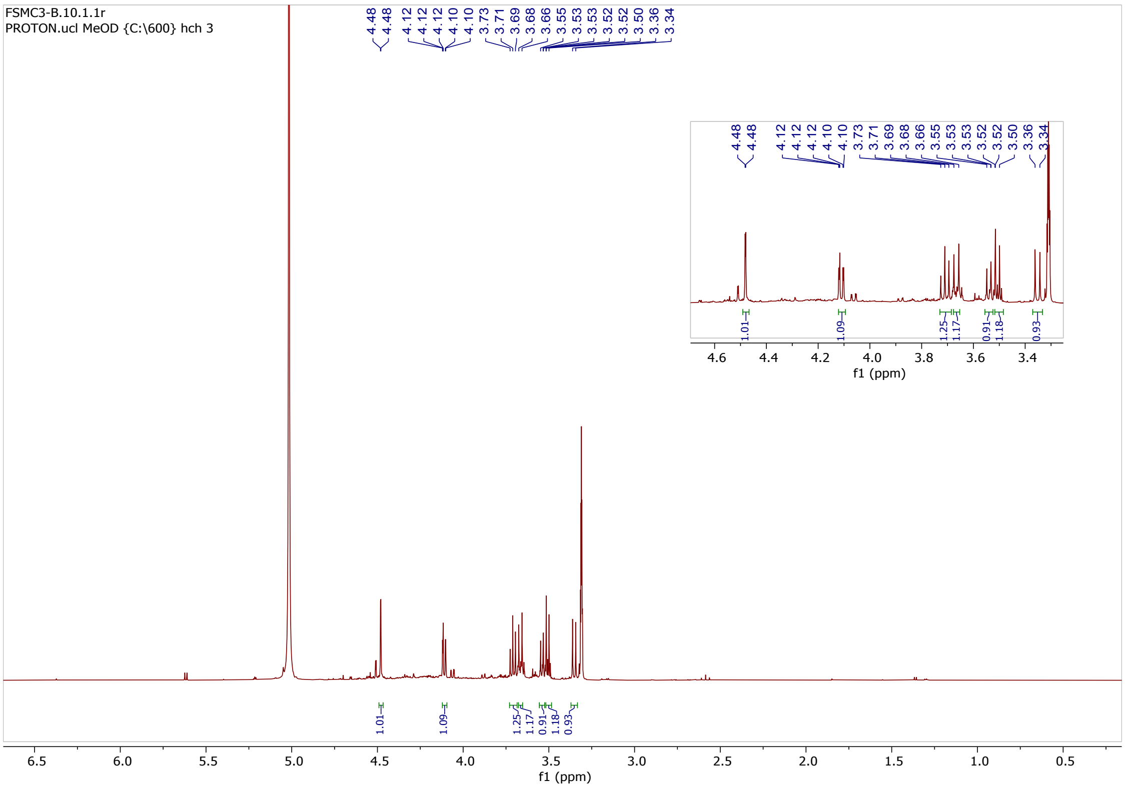

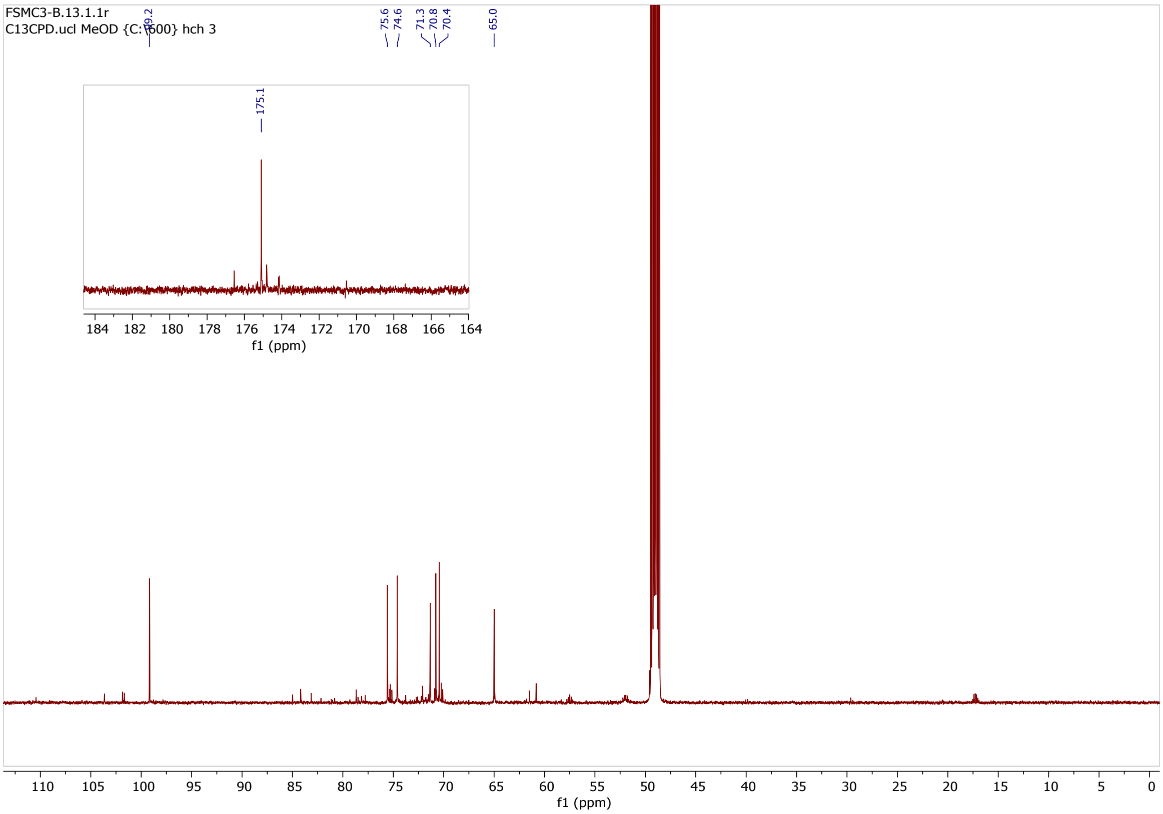

Supplementary Figure 6. ^{1}H NMR spectra of a sample of pure 7-keto-octuronic acid after 2 weeks and 4 weeks of equilibration respectively

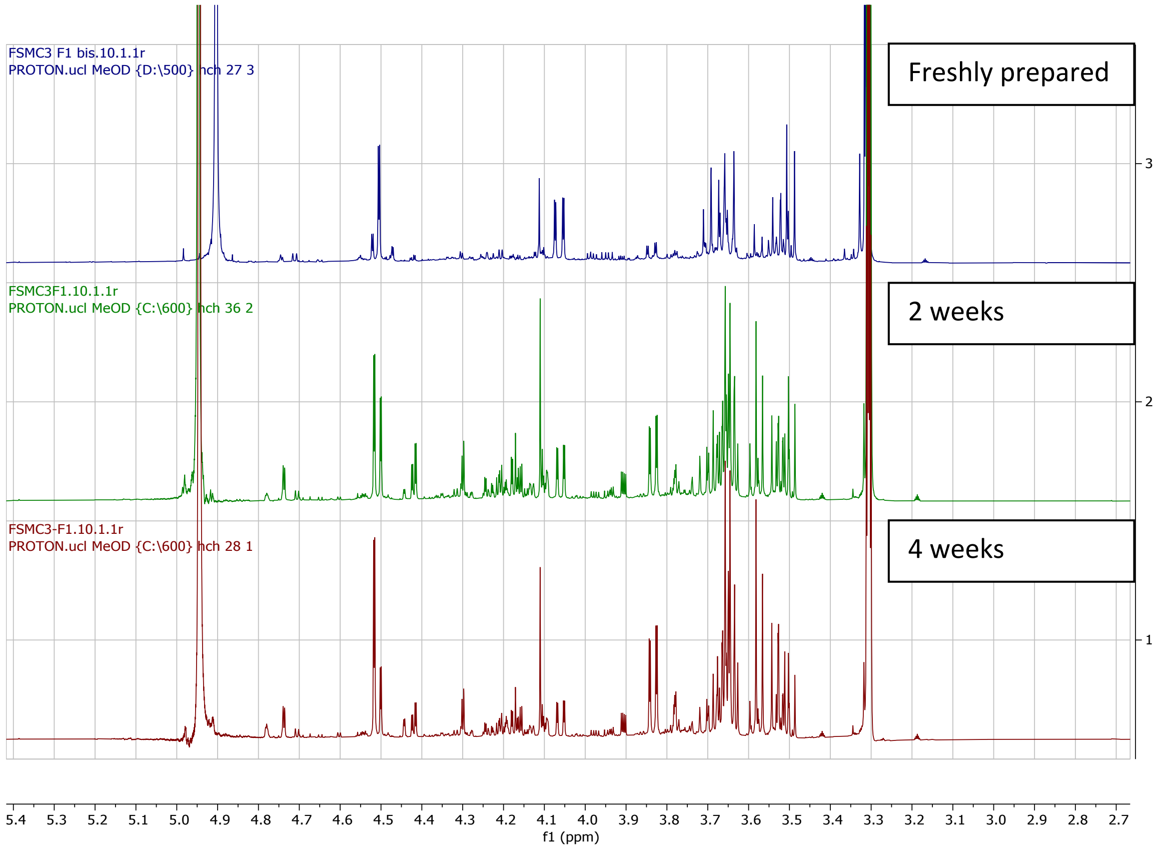

Supplementary Table 1. TKs amino acid composition (%) of TKs generated by MEGA 10 bioinformatic software. *T. maritima* (T.mar), *G. stearothermophilus* (G.ste), *E. coli* (E.col), *B. anthracis* (B.ant), *S. cerevisiae* (S.cer), *H. sapiens* (H.sap), *C. hydrogenoformans* (C.hyd) and *S. oleracea* (S.ole).

Asp	Glu	Phe	Gly	His	Ile	Lys	Leu	Met	Asn	Pro	Gln	Arg	Ser	Thr	Val	Trp	Tyr	Total
6.61	7.24	3.62	9.61	2.36	5.98	7.24	8.98	2.2	4.57	4.09	1.42	4.25	4.09	4.88	10.08	0.47	4.41	635
4.19	7.63	3.14	9.58	2.69	4.49	4.64	9.73	2.69	3.44	5.09	2.25	4.49	5.84	4.94	5.84	1.65	3.59	668
5.28	7.24	3.92	8.75	2.71	4.83	5.28	7.54	3.32	3.32	4.52	3.47	4.22	5.58	4.98	5.73	1.66	3.47	663
5.56	7.82	[illegible]	8.72	2.41	4.06	4.81	7.82	3.31	4.36	3.91	2.56	3.91	6.77	6.32	6.92	1.5	4.06	665
5	5.15	[illegible]	8.53	2.5	5.29	6.32	9.26	1.62	4.56	5.59	3.38	3.24	8.38	5.44	6.18	1.32	3.82	680
5.62	5.62	[illegible]	7.22	2.73	7.54	6.74	7.38	2.09	3.21	4.49	4.17	4.65	6.74	5.14	5.78	0.64	2.73	623
4.22	7.77	[illegible]	8.95	1.86	6.08	6.25	9.12	2.87	3.21	5.07	3.21	4.56	5.07	3.72	9.46	1.01	1.52	592
4.18	7.29	[illegible]	9.18	3.24	5.4	6.21	8.1	1.89	3.51	5.26	1.75	3.91	8.1	6.75	6.48	1.48	2.97	741
5.07	6.97	3.65	8.83	2.58	5.43	5.92	8.49	2.49	3.78	4.77	2.75	4.14	6.38	5.32	7.01	1.23	3.34	658.4

Supplementary Table 2. TKs protein sequence similarity. *T. maritima* (T.mar), G*. stearothermophilus* (G.ste), *E. coli* (E.col), *B. anthracis* (B.ant), *S. cerevisiae* (S.cer), *H. sapiens* (H.sap), *C. hydrogenoformans* (C.hyd) and *S. oleracea* (S.ole).

	**TK Similarity (%)**							
	S.cer	E.col	S.ole	G.ste	B.ant	T.mar	H.sap	C.hyd
1: S.cer	100.00	48.23	50.99	49.85	49.92	23.00	27.86	35.36
2: E.col	48.23	100.00	49.16	51.37	51.45	23.60	27.15	32.32
3: S.ole	50.99	49.16	100.00	51.29	52.88	24.50	26.31	31.79
4: G.ste	49.85	51.37	51.29	100.00	73.83	23.95	25.68	31.85
5: B.ant	49.92	51.45	52.88	73.83	100.00	24.49	24.96	31.13
6: T.mar	23.00	23.60	24.50	23.95	24.49	100.00	30.15	35.65
7: H.sap	27.86	27.15	26.31	25.68	24.96	30.15	100.00	40.24
8: C.hyd	35.36	32.32	31.79	31.85	31.13	35.65	40.24	100.00
